# 628. Pharmacokinetics, Safety and Tolerability of Co-administration of Nacubactam and β-lactams after Multiple Doses in Japanese Healthy Subjects

**DOI:** 10.1093/ofid/ofab466.826

**Published:** 2021-12-04

**Authors:** Hiroki Sato, Jun Morita, Tatsuo Miura, Masayo Sumiya, Risako Takaya, Kenichiro Kondo

**Affiliations:** Meiji Seika Pharma Co., Ltd., Tokyo, Tokyo, Japan

## Abstract

**Background:**

Increase of carbapenem-resistant Enterobacterales (CRE) is a serious problem in the clinical setting and drugs which can treat patients with CRE are still limited. Nacubactam (OP0595) is a novel diazabicyclooctane-type β-lactamase inhibitor and being developed as a standalone drug to be co-administered with cefepime or aztreonam.

**Methods:**

A randomized, double-blind multiple dose study of nacubactam in co-administration with cefepime (Cohort 1) or aztreonam (Cohort 2) in Japanese healthy subjects was performed to assess pharmacokinetics, safety, and tolerability of co-administrations of nacubactam and cefepime or aztreonam. In each cohort, 6 subjects received 2 g of nacubactam and 2 g of concomitant drug (cefepime or aztreonam) and 2 subjects received placebo (saline) intravenously over 60 minutes, three times daily every 8 hours for 7 days. Plasma samples were collected and concentrations of each drug were analyzed by liquid chromatography-mass spectrometry (LC-MS/MS). Safety and tolerability assessments included treatment-emergent adverse events (TEAEs) and the evaluation of changes from baseline in safety laboratory test results, 12-lead electrocardiograms (ECGs), vital signs, and physical examinations.

**Results:**

Profiles of C_max_, t_max_, AUC_0-8_, AUC_0-∞_ and t_1/2_ for nacubactam, cefepime and aztreonam are summarized in Table 1. Summary of C_trough_ for nacubactam, cefepime and aztreonam are summarized in Table 2. Plasma concentrations of nacubactam, cefepime and aztreonam reached the steady-state by Day 4, and the mean accumulation ratios of C_max_ and AUC_0-8_ on Day 7 to those of Day 1 were in the range of 0.91 to 1.10. As for the safety, no serious adverse event was observed in this study. There was 1 TEAE (seborrhoeic dermatitis) leading to the discontinuation in 1 subject in nacubactam/cefepime group, but it was judged as “Not related to study drug”.

Table 1. PK profiles of nacubactam and concomitant drugs on Day 1 and Day 7

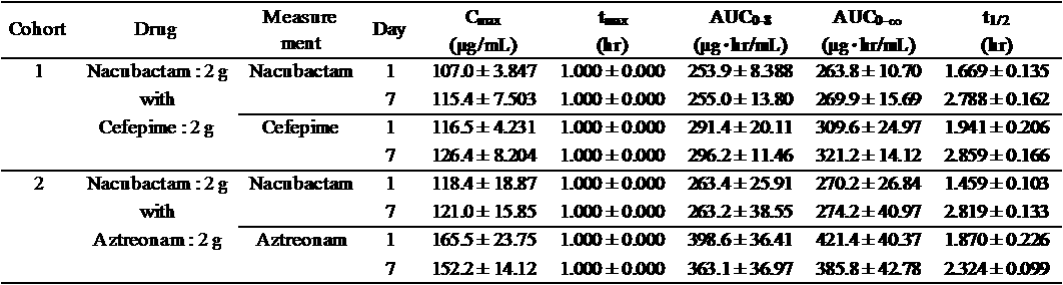

Table 2. Summary of Ctrough of nacubactam and concomitant drugs

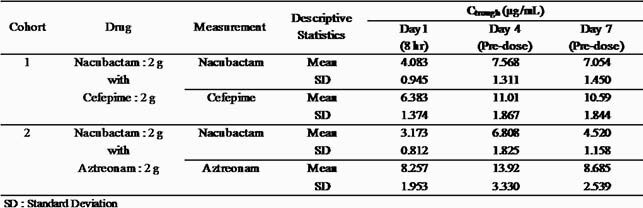

**Conclusion:**

In conclusion, no remarkable change in pharmacokinetics was observed in each drug with multiple concomitant administration for 7 days and safety and tolerability of co-administrations of nacubactam and cefepime or aztreonam were confirmed. Based on these results, nacubactam is currently under further development.

**Disclosures:**

**All Authors**: No reported disclosures

